# A virtual approach to evaluate therapies for management of multiple myeloma induced bone disease

**DOI:** 10.1002/cnm.2735

**Published:** 2015-09-02

**Authors:** Bing Ji, Paul G. Genever, Michael J. Fagan

**Affiliations:** ^1^ School of Control Science and EngineeringShandong UniversityJinan250061People's Republic of China; ^2^ Department of BiologyUniversity of YorkYorkYO10 5DDUK; ^3^ School of EngineeringUniversity of HullHullHU6 7RXUK

**Keywords:** multiple myeloma, bone disease, therapies, mathematical model

## Abstract

Multiple myeloma bone disease is devastating for patients and a major cause of morbidity. The disease leads to bone destruction by inhibiting osteoblast activity while stimulating osteoclast activity. Recent advances in multiple myeloma research have improved our understanding of the pathogenesis of multiple myeloma‐induced bone disease and suggest several potential therapeutic strategies. However, the effectiveness of some potential therapeutic strategies still requires further investigation and optimization. In this paper, a recently developed mathematical model is extended to mimic and then evaluate three therapies of the disease, namely: bisphosphonates, bortezomib and TGF‐β inhibition. The model suggests that bisphosphonates and bortezomib treatments not only inhibit bone destruction, but also reduce the viability of myeloma cells. This contributes to the current debate as to whether bisphosphonate therapy has an anti‐tumour effect. On the other hand, the analyses indicate that treatments designed to inhibit TGF‐β do not reduce bone destruction, although it appears that they might reduce the viability of myeloma cells, which again contributes to the current controversy regarding the efficacy of TGF‐β inhibition in multiple myeloma‐induced bone disease. © 2015 The Authors. *International Journal for Numerical Methods in Biomedical Engineering* published by John Wiley & Sons Ltd.

## 1 Introduction

Multiple myeloma (MM), a haematological malignancy developed in the bone marrow, is the most common cancer involving bone and the second most prevalent cancer involving blood cells 1, 2. Bone disease is a major complication of MM and is a significant cause of morbidity in MM patients. Up to 60% of MM patients suffer a fracture during the course of the disease, and MM induced bone destruction rarely heals. Recent research into MM bone disease has revealed that the interaction between MM cells and the bone microenvironment plays an important role in the development of the condition, and a ‘vicious cycle’ of myeloma development and bone destruction is established 2, 3, 4.

Currently, several therapies are proposed to treat MM‐induced bone disease including bisphosphonates, bortezomib and TGF‐β inhibition 2, 5, 6. Bisphosphonate treatments target high turnover skeletal sites, binding to the mineralized bone matrix within these sites 7, 8, 9. After their internalization by bone‐resorbing osteoclasts, bisphosphonates inhibit further osteoclast activity and bone resorption by suppressing the differentiation of osteoclast precursors into mature osteoclasts, promoting osteoclast apoptosis and disrupting osteoclast function 8, 9. Although bisphosphonates are already a first‐line treatment for MM‐induced bone disease 7, 10, further investigation is required to determine whether bisphosphonates have an anti‐tumour effect. Several preclinical and clinical studies suggest that bisphosphonates may either have a direct or indirect anti‐tumour effect 11, 12, 13, 14, 15, 16, 17, 18. However, other studies provide contradictory evidence and suggest that bisphosphonates do not improve patient mortality 19, 20, 21.

Suppression of bone‐forming osteoblasts, which can occur from the blockade of the differentiation of osteoblast precursors into mature osteoblasts, promotes the growth of myeloma cells as well as bone destruction through supporting the production of anti‐apoptotic factors and growth factors for MM cells 2, 22. Thus, it is suggested that stimulation of osteoblast differentiation may reduce tumour burden and bone destruction in MM patients 2, 23. Bortezomib, a boron‐containing compound with the potential of enhancing osteoblast proliferation and bone formation in MM patients, has therefore been proposed as a potential therapeutic for MM‐induced bone disease 24, 25.

TGF‐β is reported to contribute to the progression of MM‐induced bone disease 5. It is released with bone resorption and stimulates the production of osteoblast progenitors but inhibits the differentiation of mature osteoblasts. It therefore suppresses bone formation and indirectly promotes the progression of MM cells (immature osteoblast cells facilitate the growth and survival of MM cells, while mature cells enhance apoptosis of MM cells). Thus, the suppression of TGF‐β is proposed as a new approach to treat MM‐induced bone disease 5; however, some controversies still exist, and further investigation is required to confirm its potential.

In the paper, a mathematical model we described previously 3 was extended to simulate these three different strategies and determine their efficacies in MM (Tables 1 and 2).

**Table 1:** Definitions of the π functions. See Table 2 for definitions of RANKL, OPG, TGF‐β, PTH, IL6, SLRPs, VLA4 and VCAM1.

$\pi_{\text{act},OB_{u}}^{\text{TGFβ}}=\frac{\text{TGFβ}}{K_{D1,\text{TGFβ}}+\text{TGFβ}}$	
$\pi_{\text{rep},OB_{p}}^{\text{TGFβ}}=\frac{1}{1+\left(\text{TGFβ}/K_{D2,\text{TGFβ}}\right)}$	
$\pi_{\text{act},OC_{a}}^{\text{TGFβ}}=\frac{\text{TGFβ}}{K_{D3,\text{TGFβ}}+\text{TGFβ}}$
$\pi_{\text{act},\;OC_{p}}^{\text{RANKL}}=\frac{\text{RANKL}}{K_{D,\text{RANKL}}+\text{RANKL}}$
$\pi_{\text{act},\;MM}^{IL6}=\frac{IL6}{IL6+K_{D,IL6,MM,\text{act}}}$
$\pi_{\text{act},\;MM}^{\text{VCAM}1}=\frac{\text{VCAM}1}{\text{VCAM}1+K_{D,\text{VCAM}1,MM,\text{act}}}$
$\pi_{\text{rep},\;OB_{p}}^{\text{VCAM}1}=\frac{1}{1+\text{VCAM}1/K_{D,\text{VCAM}1,OB_{p},\text{rep}}}$
$\pi_{\text{act},\;OB_{a}}^{\text{VCAM}1}=\frac{\text{VCAM}1}{\text{VCAM}1+K_{D,\text{VCAM}1,OB_{a},\text{act}}}$
$\pi_{\text{rep},MM}^{\text{SLRPs}}=\frac{1}{1+\left(\text{SLRPs}/K_{D,\text{SLRPs},MM,\text{rep}}\right)}$
$\pi_{\text{act},\text{RANKL}}^{PTH}=\frac{PTH}{K_{D1,PTH}+PTH}$
$\pi_{\text{rep},OPG}^{PTH}=\frac{1}{1+\left(PTH/K_{D2,PTH}\right)}$
$\pi_{\text{act},\;\text{RANKL}}^{IL6}=\frac{IL6}{IL6+K_{D,IL6,\text{RANKL},\text{act}}}$
$\pi_{\text{act},IL6}^{VLA4}=\frac{VLA4}{VLA4+K_{D,VLA4,IL6,\text{act}}}$
$\pi_{\text{act},IL6}^{\text{TGFβ}}=\frac{\text{TGFβ}}{\text{TGFβ}+K_{D,\text{TGFβ},IL6,\text{act}}}$

**Table 2:** Definitions of the concentrations of RANKL, OPG, TGF‐β, PTH, IL6, SLRPs, VLA4 and VCAM1. (RANKL, receptor activator of nuclear factor kappa‐B ligand; OPG, osteoprotegerin; TGF‐β, transforming growth factor‐beta; PTH, parathyroid hormone; IL‐6, interleukin‐6; SLRP, small leucine‐rich proteoglycan; VLA‐4, very late antigen‐4; VCAM‐1, vascular cell adhesion molecule 1).

*RANKL*	$\frac{P_{\text{RANKL},d}+\beta_{\text{RANKL}}\cdot OB_{p}}{\left(1+K_{A,OPG}\cdot OPG+K_{A,\text{RANK}}\cdot \text{RANK}\right)\cdot \left(\frac{\beta_{\text{RANKL}}}{R^{\text{RANKL}}\cdot \pi_{\text{act},\text{RANKL}}^{IL6}\cdot \pi_{\text{act},\text{RANKL}}^{PTH}}+D_{\text{RANKL}}\right)}$
*OPG*	$\frac{P_{OPG,d}+\beta_{OPG}\cdot OB_{a}\cdot \pi_{\text{rep},OPG}^{PTH}}{\left(\frac{\beta_{OPG}\cdot OB_{a}\cdot \pi_{\text{rep},OPG}^{PTH}}{\text{OP}G_{\text{max}}}+D_{OPG}+D_{OPG,MM}\cdot MM\right)}$
*TGFβ*	$\frac{\alpha \cdot K_{res}\cdot OC_{a}+S_{\text{TGFβ}}}{{\tilde{D}}_{\text{TGFβ}}}$
*PTH*	$\frac{\beta_{PTH}+P_{PTH,d}\left(t\right)}{{\tilde{D}}_{PTH}}$
*IL*6	$\frac{P_{IL6,d}+\beta_{IL6}\cdot OB_{u}\cdot \pi_{\text{act},IL6}^{TGF}\cdot \pi_{\text{act},IL6}^{VLA4}}{\left(\frac{\beta_{IL6}\cdot OB_{u}\cdot \pi_{\text{act},IL6}^{TGF}\cdot \pi_{\text{act},IL6}^{VLA4}}{IL6_{\text{max}}}+D_{IL6}\right)}$
*SLRPs*	$\frac{\beta_{\text{SLRPs}}\cdot OB_{a}+P_{\text{SLRPs},d}\left(t\right)}{\left(\frac{\beta_{\text{SLRPs}}\cdot OB_{a}}{\text{SLRP}s_{\text{max}}}+{\tilde{D}}_{\text{SLRPs}}\right)}$
*VLA*4	$\frac{P_{VLA4,d}+\beta_{VLA4}\cdot MM}{\left(1+K_{A,\text{VCAM}1}\cdot \text{VCAM}1_{\text{tot}}\right)\cdot \left(\frac{\beta_{VLA4}}{R^{VLA4}}+D_{VLA4}\right)}$
*VCAM*1	$\frac{VCAM_{\text{tot}}}{1+K_{A,\text{VCAM}1}\cdot VLA4}$

## 2 Model Development

The mathematical model which simulates the pathogenesis of MM‐induced bone disease consists of the following key equations 3:

(1) $$\frac{\text{dO}B_{p}}{dt}=D_{OB_{u}}\cdot \pi_{\text{act},OB_{u}}^{\text{TGFβ}}\cdot OB_{u}-D_{OB_{p}}\cdot \pi_{\text{rep},OB_{p}}^{\text{TGFβ}}\cdot \pi_{\text{rep},OB_{p}}^{\text{VCAM}1}\cdot OB_{p}$$

(2) $$\frac{\text{dO}B_{a}}{dt}=D_{OB_{p}}\cdot \pi_{\text{rep},OB_{p}}^{\text{TGFβ}}\cdot \pi_{\text{rep},OB_{p}}^{\text{VCAM}1}\cdot OB_{p}-A_{OB_{a}}\cdot \pi_{\text{act},OB_{a}}^{\text{VCAM}1}\cdot OB_{a}$$

(3) $$\frac{\text{dO}C_{a}}{dt}\;=D_{OC_{p}}\cdot \pi_{\text{act},\;OC_{p}}^{\text{RANKL}}\cdot OC_{p}-\pi_{\text{act},OC_{a}}^{\text{TGFβ}}\cdot A_{OC_{a}}\cdot OC_{a}$$

(4) $$\frac{dMM}{dt}\;=D_{MM}\cdot \;\pi_{\text{act},\;MM}^{IL6}\cdot \pi_{\text{act},\;MM}^{\text{VCAM}1}\cdot MM\cdot \left(1-\frac{MM}{MM_{\text{max}}}\right)-A_{MM}\cdot \pi_{\text{rep},MM}^{\text{SLRPs}}\cdot MM$$

(5) $$\frac{dBV}{dt}\;=-K_{res}\cdot OC_{a}+K_{\text{form}}\cdot OB_{a}$$

where *OB_p_*, *OB_a_*, *OC_a_*, *MM* and *BV* are the populations of osteoblast precursors, active osteoblasts, active osteoclasts, active MM cells and bone volume respectively, and $\frac{\text{dO}B_{p}}{dt}$ is the variation of *OB_p_* with time, for example. Eqs. (1), (2), (3), (4), (5) describe the temporal variations in concentrations of *OB_p_*, *OB_a_*, *OC_a_*, *MM* and *BV* respectively. ‘Hill functions’ are used to represent the cellular interaction via the single ligand to receptor binding, and are denoted by π functions. The definitions of the π functions in the model equations above, and the definitions and values of the model parameters are lengthy and described in detail in the work of 3 (Open Access), but are summarized here for convenience. Table 3 contains the definitions and values of the model parameters. Any unknown parameters (i.e. those parameters where experimental data are unavailable or those which have no direct biological meaning) are calculated via a genetic algorithm (GA) as indicated in Table 3, and described in detail in 3. Briefly, because a parameter may be directly or indirectly related with one or more of the initial values of cell concentrations (listed in Table 4), e.g. $D_{OB_{u}}$ and $D_{OB_{p}}$ involve experimental data of the initial concentration of *OBp* in Table 4, these initial values are set as targets for the parameter fitting. The calculation of the model parameters is then achieved by trying different values in a domain and then selecting those that provide the best fit with the corresponding experimental data. Based on these values, the remaining unknown model parameters are then calculated according to relevant experimental data through the genetic algorithm. Thus the GA approach effectively considers all possible combinations of the unknown parameters and predicts the optimal values, as described in 3. This takes many hours on a powerful PC, potentially considering billions of combinations in its search for the optimum set. The simulation was carried using the Matlab computational software package (v7.7.0, Mathworks, Natick, USA).

**Table 3:** Definitions and values of model parameters used in the model of MM‐induced bone disease. (GA = genetic algorithm).

Parameters	Description	Value
$D_{OB_{u}}$	Differentiation rate of osteoblast progenitors	3.24e + 2/day (estimated)
$D_{OB_{p}}$	Differentiation rate of osteoblast precursors	3.67e − 1/day (estimated)
$A_{OB_{a}}$	Rate of elimination of active osteoblasts	3.00e − 1/day 32
$D_{OC_{p}}$	Differentiation rate of osteoclast precursors	1.73e − 1/day (estimated)
$A_{OC_{a}}$	Rate of elimination of active osteoclasts	1.20/day 32
*K*_*D*1,*TGFβ*_	Activation coefficient related to growth factors binding on *OB_u_*	4.28e − 4 pM (calculation by GA)
*K*_*D*2,*TGFβ*_	Repression coefficient related to growth factors binding on *OB_p_*	2.19e − 4 pM (estimated)
*K*_*D*3,*TGFβ*_	Activation coefficient related to growth factors binding on *OC_a_*	4.28e − 4 pM 32
*K*_*D*1,*PTH*_	Activation coefficient for RANKL production related to PTH binding	2.09e + 1 pM (calculation by GA)
*K*_*D*2,*PTH*_	Repression coefficient for OPG production related to PTH binding	2.21e − 1 pM 32
*K*_*D*,*TGFβ*,*IL*6,*act*_	Half‐maximal concentration of TGF‐β on promoting the production of IL‐6	1.2e − 4 pM (calculation by GA)
*K*_*D*,*IL*6,*RANKL*,*act*_	Half‐maximal concentration of IL6 on promoting the production of RANKL	0.2 pM (calculation by GA)
*K*_*D*,*RANKL*_	Activation coefficient related to RANKL binding to RANK	4.12e + 1 pM (estimated)
α	TGF‐β content stored in bone matrix	1.00 pM/% 32
${\tilde{D}}_{\text{TGFβ}}$	Rate of degradation of TGF‐β	2.00e + 2/day 33
β_PTH_	Rate of synthesis of systemic PTH	9.74e + 2 pM/day 34
${\tilde{D}}_{PTH}$	Rate of degradation of PTH	3.84e + 2/day 34
β_IL6_	Rate of synthesis of IL6 per cell	1.20e + 7/day 35, 36
D_IL6_	The degradation rate of IL6	4.99e + 1/day 37
IL6_max_	The maximum concentration of IL‐6	8.04e − 1 pM 38
β_OPG_	Minimum rate of production of OPG per active osteoblast	5.02e + 6/day (estimated)
${\tilde{D}}_{OPG}$	Rate of degradation of OPG	4.16/day 39
OPG_max_	Maximum possible OPG concentration	7.98e + 2 pM 40
β_RANKL_	Production rate of RANKL per cell	8.25e + 5/day (estimated)
${\tilde{D}}_{\text{RANKL}}$	Rate of degradation of RANKL	4.16/day 41
*R* ^*RANKL*^	Maximum number of RANKL on the surface of each osteoblastic precursor	3.00e + 6 32
RANK	Fixed concentration of RANK	1.28e + 1 pM 32
*K*_*A*,*OPG*_	Association rate constant for RANKL binding to OPG.	5.68e − 2/pM 42
*K*_*A*,*RANK*_	Association rate constant for RANKL binding to RANK.	7.19e − 2/pM 42
*K*_*res*_	Relative rate of bone resorption (normalized with respect to normal bone resorption)	2.00e + 2%/(pM day) 43
*K*_*form*_	Relative rate of bone formation (normalized with respect to normal bone resorption)	3.32e + 1%/(pM day) (calculation by GA)
*D*_*MM*_	MM proliferation controlled by IL‐6 and BMSC‐MM adhesion	5.50e − 2/day (estimated)
*A*_*MM*_	Rate of elimination of active MM cells	2.00e − 3/day 44
*MM*_*max*_	Maximum possible MM cell concentration	1.98 pM 45
*K*_*D*,*VCAM*1,*MM*,*act*_	Half‐maximal concentration of *VLA‐4* on promoting the MM cells production	1.5667e − 4/pM (calculation by GA)
*K*_*D*,*VLA*4,*IL*6,*act*_	Half‐maximal concentration of *VLA* − 4*VLA* − 4 on promoting the IL‐6 production	1.88e + 4/pM (calculation by GA)
*K*_*D*,*IL*6,*MM*,*act*_	Half‐maximal concentration of *IL‐6* on promoting the MM cells production	1.2151e − 5 pM (calculation by GA)
*K*_*D*,*SLRPs*,*MM*,*rep*_	Half‐maximal concentration of *SLRPs* on promoting the MM cells production	1.306e + 9 pM (calculation by GA)
$K_{D,\text{VCAM}1,OB_{p},\text{rep}}$	Half‐maximal concentration of *VCAM‐1* on repressing the differentiation of *OB*_*p*_	1.4e − 1 pM (calculation by GA)
$K_{D,\text{VCAM}1,OB_{a},\text{act}}$	Half‐maximal concentration of *VCAM‐1* on promoting the apoptosis of OB_a_	2.2e − 1 pM (calculation by GA)
β_VLA4_	Rate of synthesis of *VLA‐4* per cell	2.04e + 6/day (estimated)
${\tilde{D}}_{VLA4}$	Rate of degradation of *VLA‐4*	1.5/day (estimated)
*R* ^*VLA*4^	Maximum number of *VLA‐4* expressed on the surface of MM cells	5.6e + 4 46
VCAM1_tot_	Total concentration of *VCAM‐1*	1.92 pM 46
K_A,VCAM1_	The association rate for *VLA‐4* binding to *VCAM‐1*	8.3e − 2/pM 47
D_OPG,MM_	The degradation rate of *OPG* by MM cells	4.16/(pM day) (estimated)

**Table 4:** The initial values of cell concentrations in the model.

Variables	Description	Values
*OB*_*u*_	Uncommitted osteoblastic progenitors	3.27e − 6 pM
*OB*_*p*_	Osteoblast precursors	7.67e − 4 pM
*OB*_*a*_	Active osteoblasts	6.39e − 4 pM
*OC*_*p*_	Osteoclastic precursors	1.28e − 3 pM
*OC*_*a*_	Active osteoclasts	1.07e − 4 pM
MM	Active MM cells	3.26e − 1 pM

Notes: MM cell concentration is at day 51; other cell concentrations are at day 1.

### 2.1 Modelling bisphosphonates treatment

Bisphosphonate treatments inhibit bone resorption by suppressing the differentiation of mature osteoclasts as well as promoting the apoptosis of osteoclasts. Eq. (3) describes the variation of osteoclasts with time for patients with MM‐induced bone disease. In order to investigate the efficacy of biphosphonate treatments against the disease, a parameter F.Bi, representing the degree that the bisphosphonates inhibit bone resorption, is added in Eq. (3), and the new equation is as follows:

(6) $$\frac{\text{dO}C_{a}}{dt}\;=D_{OC_{p}}\cdot \pi_{\text{act},\;OC_{p}}^{\text{RANKL}}\cdot OC_{p}\cdot F.\text{Bi}-\pi_{\text{act},OC_{a}}^{\text{TGFβ}}\cdot A_{OC_{a}}\cdot OC_{a}\cdot \left(1+\left(1-F.\text{Bi}\right)\right)\text{.}$$

The value of parameter F.Bi is in the range of [0, 1] and is negatively correlated to the concentration of bisphosphonate during the treatment, thus a small value of F.Bi corresponds to a large dosage of bisphosphonate, which would produce a corresponding decrease in the differentiation rate of mature osteoclasts and thus osteoclast apoptosis is stimulated. For example, when F.Bi is set as 0.7, the differentiation rate of active osteoclasts is decreased to 70% (0.7), while the apoptosis of osteoclasts increases by 30% (1 − F.Bi).

### 2.2 Modelling bortezomib treatment

Bortezomib stimulates osteoblast proliferation and bone formation in MM patients, which can potentially inhibit the growth of myeloma cells as well as bone destruction. Eq. (2) represents the temporal variation of osteoblasts under the condition of MM‐induced bone disease. In order to simulate bortezomib treatment, a parameter F.Bo, which represents the degree by which osteoblast differentiation is promoted, is introduced to extend Eq. (2), and the new equation is as follows:

(7) $$\frac{\text{dO}B_{a}}{dt}=D_{OB_{p}}\cdot \pi_{\text{rep},OB_{p}}^{\text{TGFβ}}\cdot \pi_{\text{rep},OB_{p}}^{\text{VCAM}1}\cdot OB_{p}\cdot F.\text{Bo}-A_{OB_{a}}\cdot \pi_{\text{act},OB_{a}}^{\text{VCAM}1}\cdot OB_{a}\text{.}$$

The value of parameter F.Bo is in the range of (1, + ∞), and is positively related to the dosage of bortezomib during the treatment. For example, when F.Bo is set to 2.0, osteoblast activity is increased two‐fold.

### 2.3 Modelling TGF‐β inhibition treatment

TGF‐β stimulates the production of osteoblast progenitors while inhibiting the differentiation of mature osteoblasts as shown in Eqs. (1) and (2), and thus the inhibition of TGF‐β indirectly suppresses the progression of MM cells, because immature osteoblast cells facilitate the growth and survival of MM cells, while mature cells enhance apoptosis of MM cells. In addition, TGF‐β can also promote the apoptosis of active osteoclasts as shown in Eq. (3). In the model of 3, the concentration of TGF‐β is defined as follows:

(8) $$\text{TGFβ}=\frac{\alpha \cdot K_{res}\cdot OC_{a}+S_{\text{TGFβ}}}{{\tilde{D}}_{\text{TGFβ}}}\text{.}$$

The definitions and values of parameters in Eq. (8) are included in Table 3. In order to examine the potential of TGF‐β inhibition treatment against MM‐induced bone disease, a parameter F.T, which describes the degree by which TGF‐β is suppressed, is added into Eq. (8), so that the concentration of TGF‐β is updated to:

(9) $$\text{TGFβ}=\frac{\alpha \cdot K_{res}\cdot OC_{a}+S_{\text{TGFβ}}}{{\tilde{D}}_{\text{TGFβ}}}\cdot F.T$$

where the value of parameter F.T is in the range of [0, 1], and is negatively related to the concentration of TGF‐β during the treatment; for example, an F.T value of 0.9 represents a reduction in TGF‐β concentration to 90% of its normal value. As a result, model Eqs. (1), (2), (3), which contain TGF‐β, are all updated as well.

## 3 Simulation and Analysis

In all the following simulations, MM cell invasion is assumed to occur at day 51 with the different interventions applied at day 301, once the MM and bone cell populations (*OB_p_*, *OB_a_* and *OC_p_*) have stabilized again to their final steady state value (to 578%, 293%, 199% and 249% of their values on day 50). Because the actual relationships between parameters (F.Bi, F.Bo and F.T) and equivalent drug dosages are currently not known, sample values of the parameters are investigated initially, together with further simulations to determine the sensitivity of the results to those values.

### 3.1 Simulation of bisphosphonates treatment

**Figure 1:**
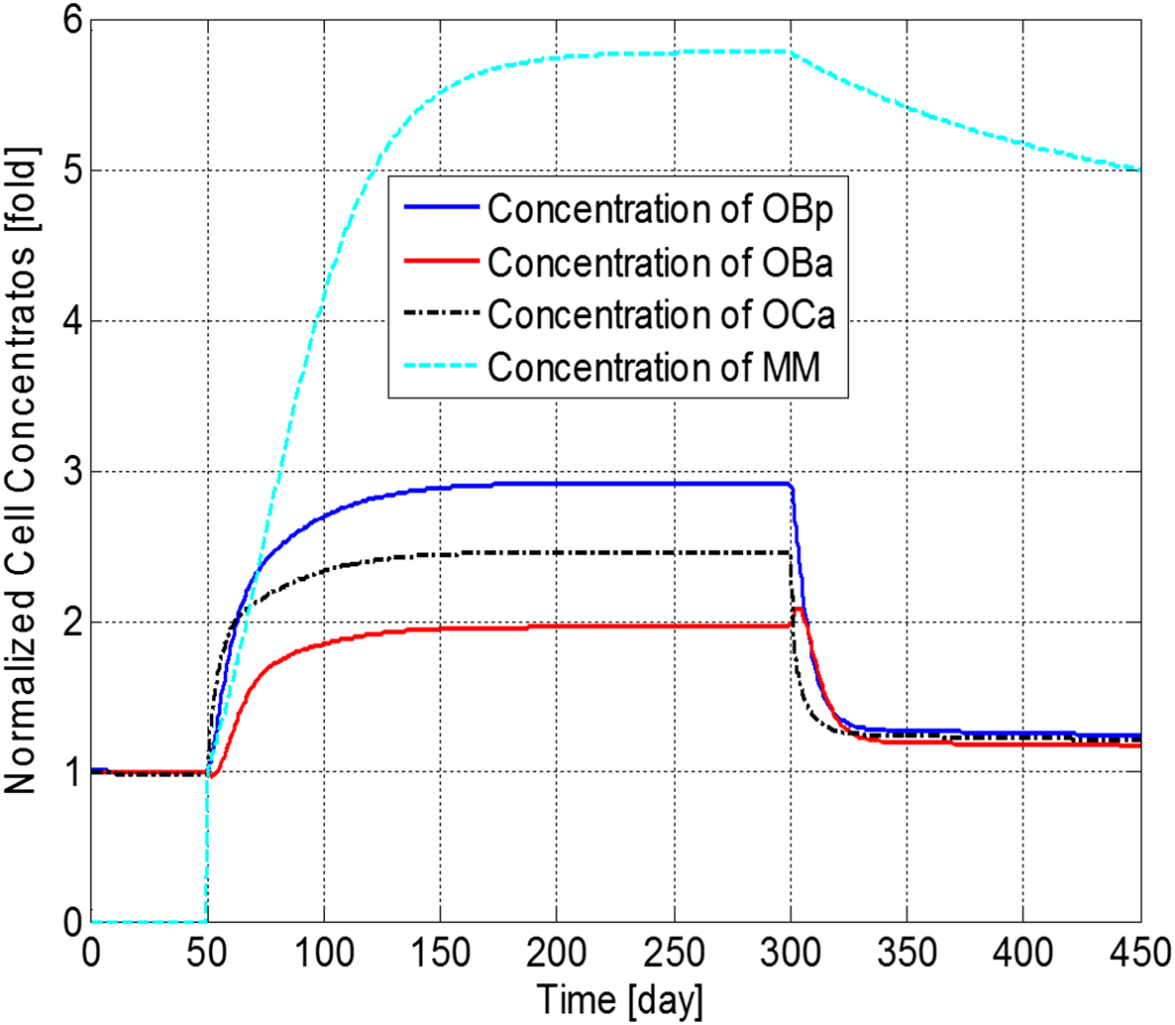
The variation of normalized cell concentrations with respect to their initial value during different periods. Days 1 to 50: normal period; days 51 to 300: invasion of MM cells; and from day 301: intervention with bisphosphonate therapy.

**Figure 2:**
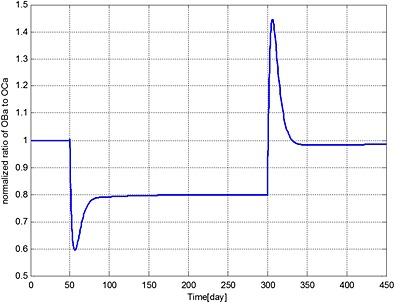
The variation of normalized ratio of OBa:OCa with respect to its initial value during different periods. Days 1 to 50: normal period; days 51 to 300: invasion of MM cells; and from day 301: intervention with bisphosphonate therapy.

**Figure 3:**
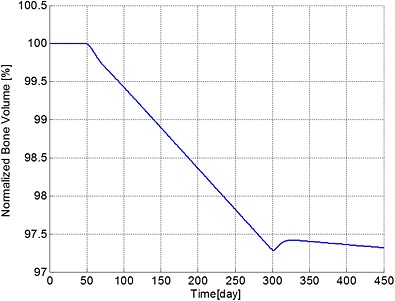
The variation of normalized bone volume with respect to its initial value during different periods. Days 1 to 50: normal period; days 51 to 300: invasion of MM cells; and from day 301: intervention with bisphosphonate therapy.

Figures 1 to 3 demonstrate how a bisphosphonate therapy, with F.Bi = 0.7, might influence cell concentrations and bone volume. Figure 1 shows a rapid increase in the population of MM cells after their initial appearance at day 51. Treatment at day 301 then leads to a reduction in peak concentration of 16% by day 450 and a continued decrease until a stable value is achieved at day 1743 (not shown) of 4.43 times the original value (at day 51). Bone cell concentrations similarly increase with the presence of the MM cells, but quickly return to a new stable state of typically 110% of their normal values (i.e. before the invasion of the myeloma cells).

The ratio of bone cells, OBa:OCa, is predicted to decrease quickly to 80% of its initial value after invasion of the MM cells (Figure 2), leading to a linear decrease in bone volume until application of the bisphosphonate therapy at day 301 (Figure 3). At this point, the OBa:OCa ratio peaks but quickly returns to 98% of its initial value, resulting in a significant slowdown in bone destruction as shown in Figure 3 after day 301.

**Figure 4:**
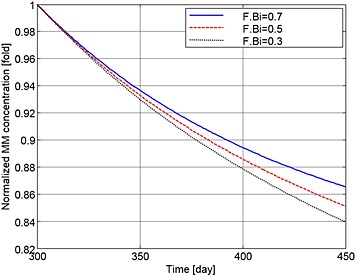
The variation of normalized MM cell concentration with respect to the value at day 300 after use of the bisphosphonates therapy with different values of F.Bi.

**Figure 5:**
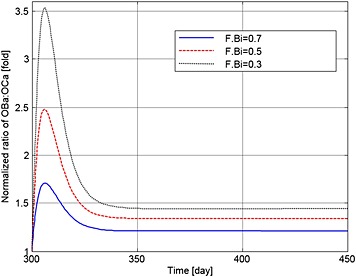
The variation of normalized ratio of OBa:OCa with respect to the value at day 300 after use of the bisphosphonate therapy with different values of F.Bi.

**Figure 6:**
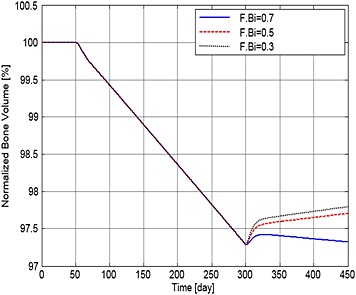
The variation of normalized bone volume with respect to its initial value after use of the bisphosphonate therapy with different values of F.Bi.

Figures 4 to 6 show how MM cell concentration, bone volume and OBa:OCa ratio vary for sample F.Bi bisphosphonate inhibition values (of 0.7, 0.5 and 0.3) for the same treatment strategy. In relation to their peak values at day 300, MM cell population decreases to 86.8%, 85.2% and 84% (Figure 4), and OBa:OCa ratio increases to 123%, 134% and 144% when F.Bi is set to 0.7, 0.5 and 0.3 respectively (Figure 5). As illustrated in Figure 6, when F.Bi is set as 0.7, bone destruction continues, although its rate is decreased markedly, because of the increased OBa:OCa ratio. However when F.Bi is set to 0.5 or 0.3, bone destruction is halted and bone volume begins to increase again. Thus, the simulation results suggest that a smaller value of F.Bi produces a more significant inhibition of MM cell viability and bone destruction.

### 3.2 Simulation of bortezomib treatment

**Figure 7:**
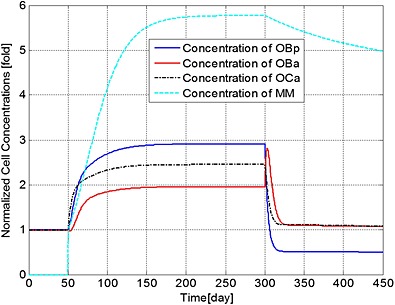
The variation of normalized cell concentrations with respect to their initial values during different periods. Days 1 to 50: normal period; days 51 to 300: invasion of MM cells; and from day 301: intervention with bortezomib therapy.

**Figure 8:**
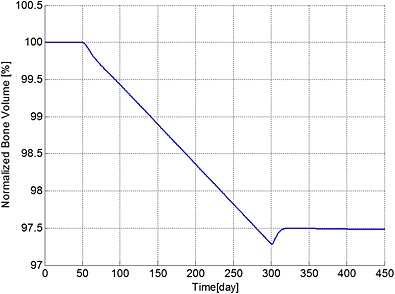
The variation of normalized bone volume with respect to its initial value during different periods. Days 1 to 50: normal period; days 51 to 300: invasion of MM cells; and from day 301: intervention with bortezomib therapy.

**Figure 9:**
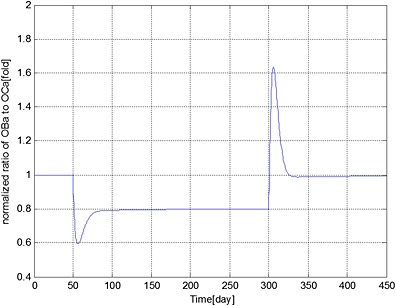
The variation of normalized ratio of OBa:OCa with respect to its initial value during different periods. Days 1 to 50: normal period; days 51 to 300: invasion of MM cells; and from day 301: intervention with bortezomib therapy.

Simulation results for the bortezomib therapy, applied at day 301 with F.Bo set to 2.2, are shown in Figures 7 to 9, and again present the variations in cell concentrations, bone volume and the ratio of OBa:OCa. The bortezomib causes a decrease in the population of MM cells (Figure 7), with concentrations of OBp, OBa and OCa also decreasing and approaching new equilibrium points by day 450. For OBa and OCa these levels are near their initial values before the invasion of MM cells, but OBp values are reduced by 51%. Figure 8 shows that further MM‐induced bone loss is prevented after a short period of fluctuation through the intervention with bortezomib, while the OBa:OCa ratio (shown in Figure 9) again undergoes a short period of fluctuation and then returns to a level similar to its original value without the myeloma cells. This explains the termination or inhibition of MM‐induced bone loss because of bortezomib.

**Figure 10:**
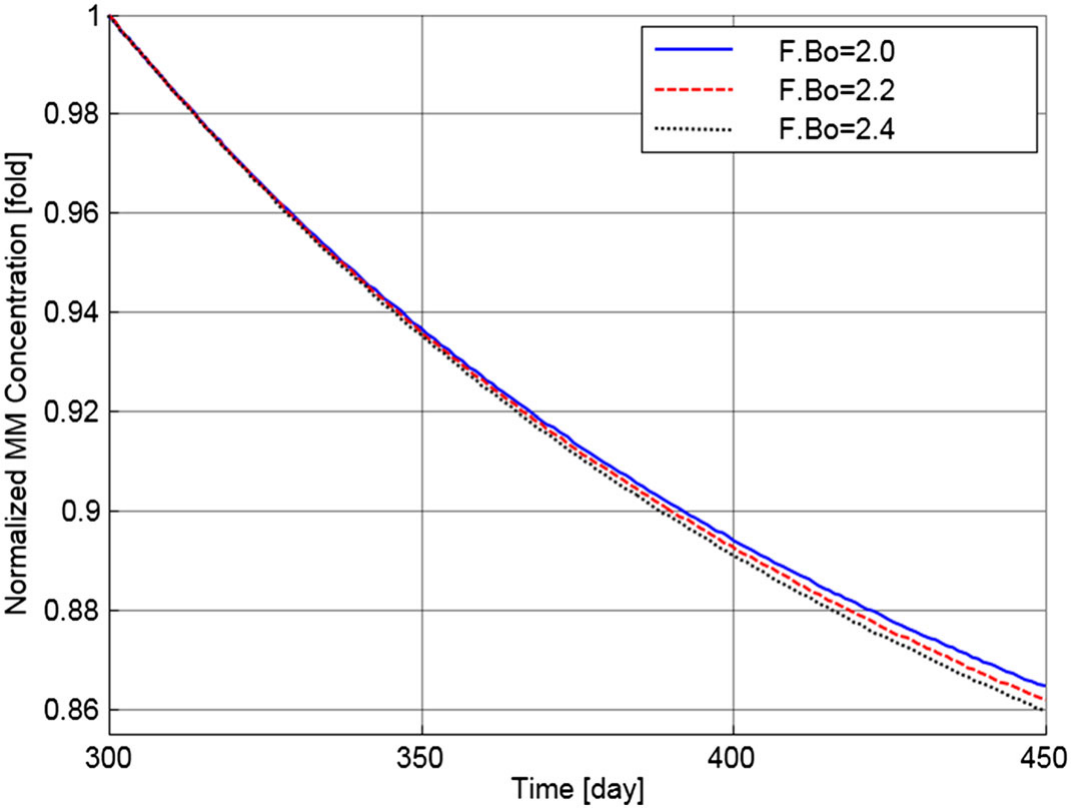
The variation of normalized MM cell concentration with respect to the value at day 300 after use of the bortezomib therapy with different values of F.Bo.

**Figure 11:**
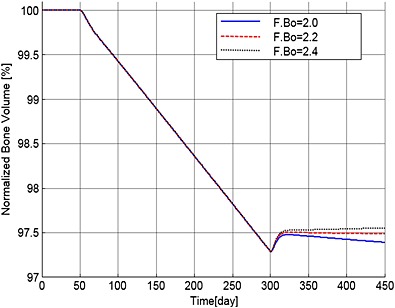
The variation of normalized bone volume with respect to its initial value after use of the bortezomib therapy with different values of F.Bo.

**Figure 12:**
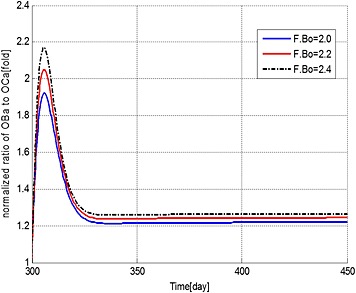
The variation of normalized ratio of OBa:OCa with respect to the value at day 300 after use of the bortezomib therapy with different values of F.Bo.

Sensitivity of the simulations to the value of F.Bo is explored in Figures 10 to 12, which show the variations in the output data with F.Bo values of 2.0, 2.2 and 2.4. Overall the simulations are not particularly sensitive to this level of variation. MM cell population decreases to 86.4%, 86.2% and 86.0%, bone volume decreases to 97.4%, 97.5% and 97.6% and OB_a_:OC_a_ ratio increases to 122.1%, 124.5% and 126.7%, respectively. In Figure 11, when F.Bo equals 2.0, MM‐induced bone loss continues although its rate is greatly reduced, because of the increased OBa:OCa ratio (Figure 12). When F.Bo is 2.2 and greater, a near zero or positive bone balance is achieved after the bortezomib therapy. The results suggest that the degree of reduction in MM cell viability and mitigation of bone destruction are both positively related to the value of F.Bo.

### 3.3 Simulation of TGF‐β inhibition treatment

**Figure 13:**
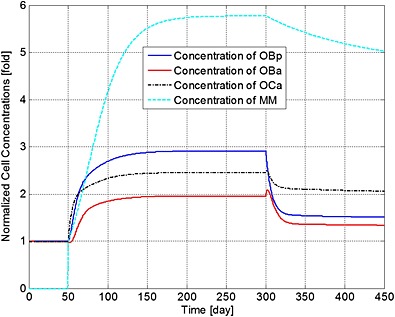
The variation of normalized cell concentrations with respect to their initial values during different periods. Days 1 to 50: normal period; days 51 to 300: invasion of MM cells; and from day 301: intervention with TGF‐β therapy.

**Figure 14:**
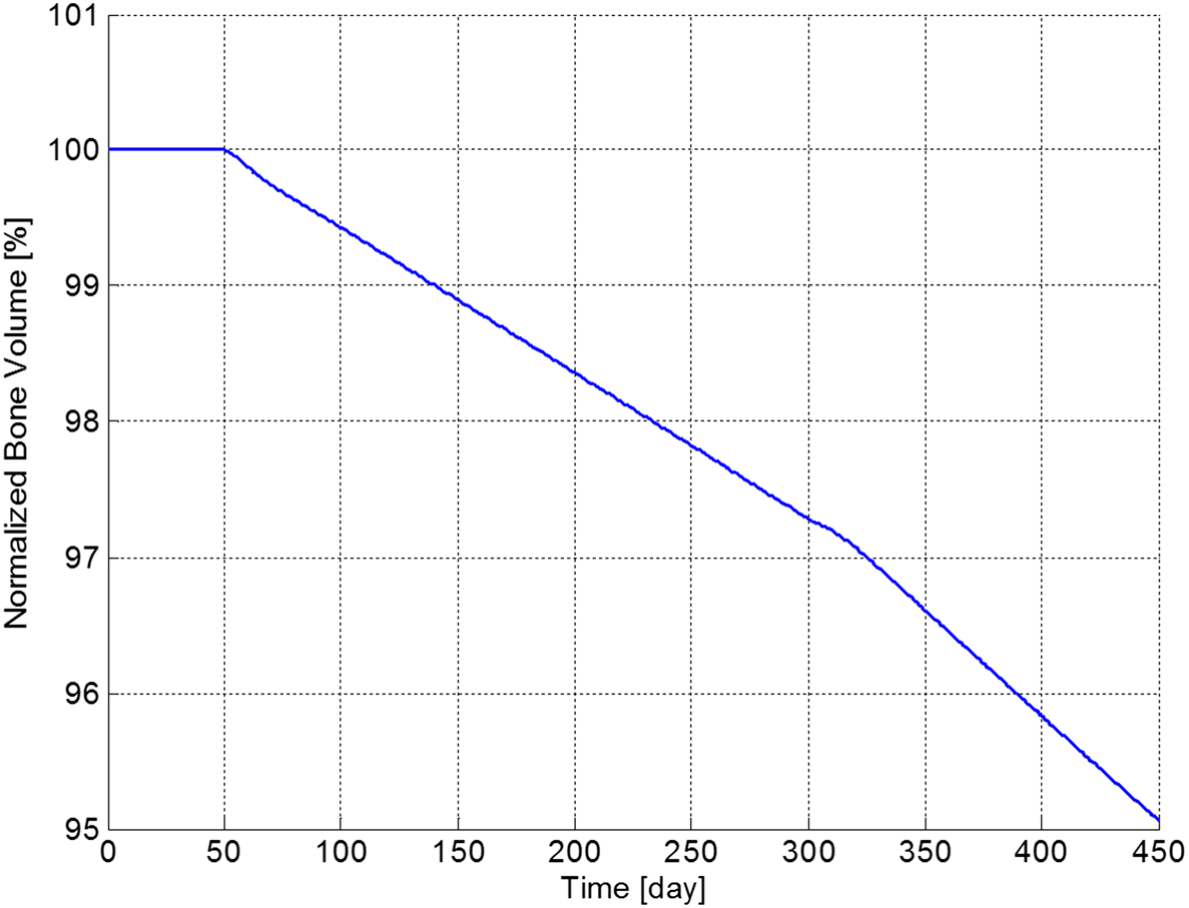
The variation of normalized bone volume with respect to its initial value during different periods. Days 1 to 50: normal period; days 51 to 300: invasion of MM cells; and from day 301: intervention with TGF‐β therapy.

**Figure 15:**
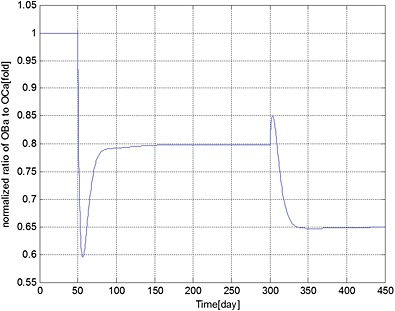
The variation of normalized ratio of OBa:OCa with respect to its initial value during different periods. Days 1 to 50: normal period; days 51 to 300: invasion of MM cells; and from day 301: intervention with TGF‐β therapy.

The variations in cell concentrations, bone volume and the ratio of OBa:OCa after the intervention of TGF‐β therapy (from day 301 with F.T set to 0.7) are presented in Figures 13 to 15. TGF‐β suppression leads to a decline in MM cell population and bone cell concentrations, with the reduction of 13.0% in MM cell numbers (at day 450) suggesting that the tumour burden can be reduced through the inhibitionof TGF‐β. However, the MM‐induced bone destruction actually increases after the TGF‐β therapy (Figure 14). This increase in bone loss can be explained by the 18.7% decrease in OBa:OCa ratio, compared to the value at day 300, caused by the TGF‐β therapy (as shown in Figure 15).

**Figure 16:**
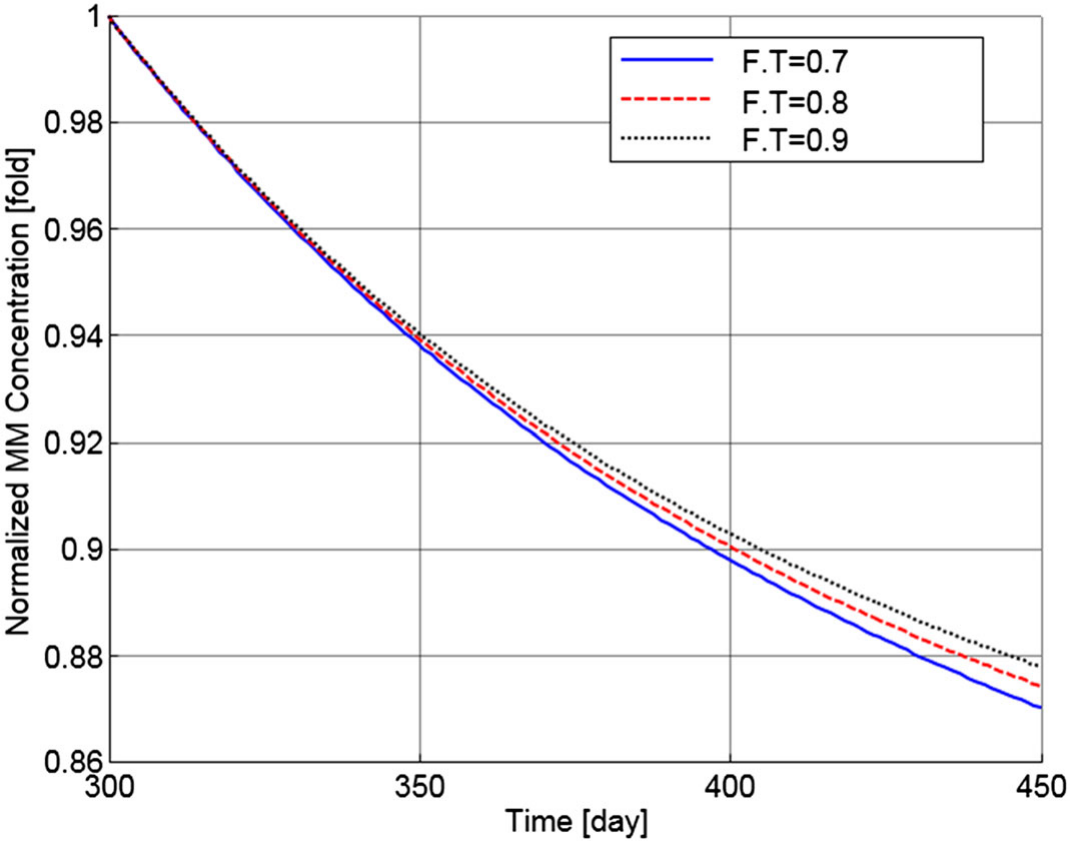
The variation of normalized MM cell concentration with respect to the value at day 300 after use of the TGF‐β therapy with different values of F.T.

**Figure 17:**
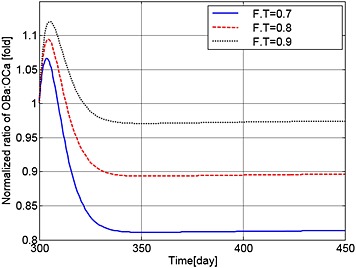
The variation of normalized ratio of OBa:OCa with respect to the value at day 300 after use of the TGF‐β therapy with different values of F.T.

**Figure 18:**
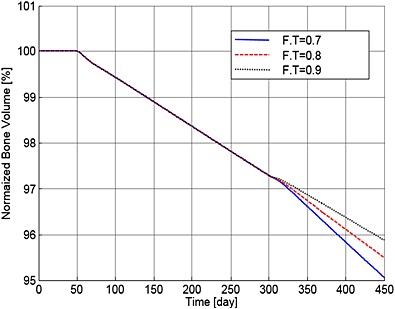
The variation of normalized bone volume with respect to its initial value after use of the TGF‐β therapy with different values of F.T.

Finally, Figures 16 and 17 show the variations in the output data with different values of F.T (of 0.7, 0.8 and 0.9). As a result, the population of MM cells decreases to 87.0%, 87.4% and 87.8%, and the OBa:OCa ratio decreases to 81.3%, 89.6% and 97.41% of the normal value, respectively. The decrease in the ratio of active osteoblasts to osteoclasts (observed with all applications of TGF‐β) leads to continued bone loss after treatment, with the lowest levels of TGF‐β leading to the greatest loss of bone volume (Figure 18).

## 4 Discussion

In this paper, a mathematical model first described in 3 was extended to simulate and evaluate three different therapeutic approaches to manage MM‐induced bone disease. Full details of the basic model and its validation are described in length in that publication, and are not repeated here. The therapies investigated are: bisphosphonates, bortezomib and TGF‐β inhibition, and their effects on MM and bone cell populations and bone volume are considered.

Bisphosphonates are used widely in the management of MM‐induced bone disease, and are able to inhibit osteoclast activity and bone resorption. However, the degree to which it affects MM cell viability and has an anti‐tumour effect is not clear. The model simulation suggests that bisphosphonate therapy can not only suppress bone loss, but also reduce MM cell population. This is confirmed by published data that reporting that bisphosphonates suppress MM‐induced bone destruction 8, 9. It is should be noted that direct anti‐tumour effects from the bisphosphonate are not considered in the model; thus, the decreased tumour burden is due solely to the inhibited osteoclast activity, indicating that bisphosphonate therapy has an indirect anti‐tumour effect. This finding agrees with experimental observations that a decrease in osteoclast activity inhibits proliferation of MM cells 6, 26. The underlying mechanism for the indirect effect of bisphosphonate treatment lies in the fact that bisphosphonate therapy suppresses bone resorption, and thus results in a decrease in TGF‐β release, which then inhibits the proliferation of MM cells by suppressing IL‐6 secretion, because IL‐6 promotes the proliferation of MM cells 5, 8, 27, 28.

Osteoblast suppression occurring in MM patients facilitates the growth of MM cells and bone loss; therefore, bortezomib, which can enhance osteoblast proliferation, is suggested as a potential therapeutic intervention for MM. In its simulation here, bortezomib therapy is indeed shown to be effective in the management of MM‐induced bone disease through its action to decrease the viability of MM cells while limiting MM‐induced bone destruction. The inhibition of MM cells by the bortezomib therapy agrees with the experimental finding that increased osteoblast proliferation is able to reduce tumour burden in MM patients 23, 29, 30. Thus, the stimulation of osteoblast activity with therapies, such as bortezomib, can inhibit or even stop bone destruction as well as the tumour burden, and is an effective therapy for MM patients.

Because TGF‐β can indirectly promote the progression of MM cells, its inhibition is also suggested as a possible treatment for MM‐induced bone disease 5. However, although the model simulation indicates that this approach can lead to a decrease in MM cell viability, the MM‐induced bone loss is not inhibited and can become worse. In addition to the effect of TGF‐β on osteoblasts, TGF‐β can also inhibit osteoclasts by promoting their apoptosis 31, and TGF‐β inhibition would unavoidably lead to an increase in osteoclast activity and resultant bone resorption. This explains the increased bone loss resulting from TGF‐β inhibition. Therefore the MM management through inhibition of TGF‐β treatment does not appear to be effective using our modelling techniques.

The relationships between the treatment parameters (F.Bi, F.Bo and F.T) and equivalent drug dosages are currently not known; hence simulations to determine the sensitivity of the results to the values have been undertaken. The different treatment options can be clearly seen to work in different ways, and notwithstanding the uncertainty in the parameter values, all three simulations show qualitative agreement with the available clinical data, providing some degree of confidence in the model. Clearly further work and quantitative clinical data is required to confirm the parameter values and validate the model and its use in this application. Until then, it is hoped that this paper can serve as a virtual evaluation tool, which can be used to suggest new therapies or combinations of therapies and to explore the possible effectiveness of new therapeutic approaches before embarking on expensive clinical trials.
